# Case report: Dupilumab therapy for alopecia areata in a 4-year-old patient resistant to baricitinib

**DOI:** 10.3389/fmed.2023.1253795

**Published:** 2023-10-09

**Authors:** Lu Cai, Yi Wei, Min Zhao, Jia Zhuo, Xiao Tao, Mao Lin

**Affiliations:** ^1^Department of Dermatology, Chongqing Hospital of Traditional Chinese Medicine, Chongqing, China; ^2^Chongqing Medical University, Chongqing, China; ^3^Department of Dermatology, West China Hospital, Sichuan University, Chengdu, Sichuan, China

**Keywords:** alopecia areata, dupilumab, resistant to baricitinib, pediatric patients, Th1, Th2

## Abstract

Alopecia areata (AA) is a non-scarring hair loss disorder. Alopecia totalis (AT) and alopecia universalis (AU) are the severe subtypes of AA. Age of onset before 6 years of age, disease duration of more than 1 year, and extensive alopecia involving more than 50% of the scalp (including AT or AU) suggest a poorer prognosis. Topical corticosteroids are the preferred first-line treatment for pediatric AA. While some treatments, such as intralesional corticosteroids, systemic steroids, contact immunotherapy with squaric acid dibutyl ester, and JAK inhibitors, showed efficacy in adults with AA, their safety profiles limit their use in pediatric AA patients. Dupilumab is a biologic that effectively addresses the patho-physiology of Th2 allergic diseases, and treats atopic diseases by inhibiting the helper Th2 immune axis. AA has been reported to be significantly improved with dupilumab for atopic dermatitis (AD) in children and adults. We report hair regrowth over all of the scalp, eyebrows, and eyelashes after 10 months of dupilumab therapy in a 4-year-old AU patient resistant to baricitinib.

## Background

Alopecia universalis (AU), one of the severe subtypes of alopecia areata (AA), is a disease with a poor prognosis that is prone to recurrence and that seriously affects the mental health of patients (1). AA is an autoimmune disease that is mainly mediated by Th1 cell pathway activity. It has been reported that AA may be associated with the systemic dysregulation of Th1 (IL-2, IFN-γ, TNF, and IL-12), Th2 (IL-6), and Th17 (IL-17 and IL-21) cytokines by detecting the relevant cytokines in the patients' serum (2). AA pathogenesis is associated with the production of IFN-γ through the JAK1 and JAK2 pathways, which stimulate IL-15 production by follicular epithelial cells (1). In addition to Th1, new data support that atopic background and Th2-skewing may play a role in AA (3–5). Available treatments for AA include corticosteroids, immunomodulators, and JAK inhibitors. In phase 3 trials of the JAK inhibitor baricitinib for AA, baricitinib has achieved excellent efficacy and has been approved by the Food and Drug Administration (FDA) for the treatment of severe AA (6). However, some patients still have poor response rates. A phase 2a randomized clinical trial confirmed that dupilumab is effective in patients with AA, both with and without concomitant AD. Patients with high levels of IgE have a higher response rate to dupilumab therapy (7). Dupilumab may be a promising therapy for JAK inhibitor-resistant severe AA patients, especially pediatric patients.

## Case presentation

**Figure 1:**
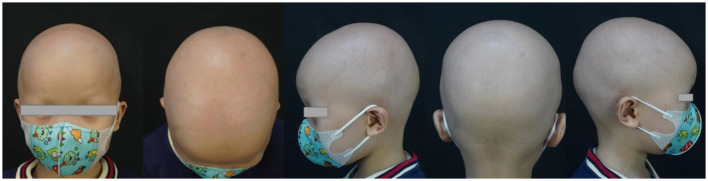
Before dupilumab therapy.

**Figure 2:**
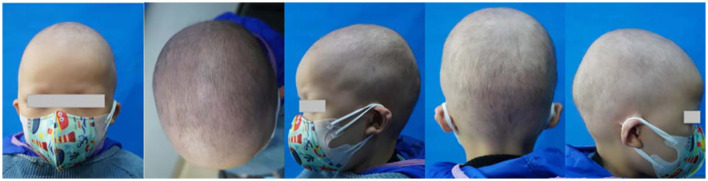
After 3 months of dupilumab therapy.

**Figure 3:**
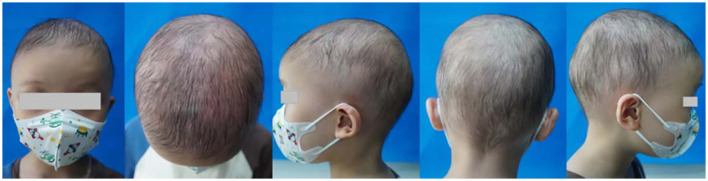
After 10 months of dupilumab therapy.

We report hair regrowth over all of the scalp, eyebrows, and eyelashes after 10 months of dupilumab therapy in a 4-year-old AU patient resistant to baricitinib. The patient suffered from AU for 3 years. He also had allergic rhinitis but had no history of other allergic and autoimmune diseases, such as asthma, eczema, systemic lupus erythematosus, or a relevant disease in his family history. He did not show abnormal damage to the nails. There were no abnormalities in the routine blood test, IgE levels, or allergen test. The patient intermittently used topical corticosteroids, such as 0.1% mometasone furoate cream and 0.05% halometasone cream, with occlusion for approximately 2 years. The SALT score was 0, with no hair regrowth in the eyebrows and eyelashes and no abnormal damage to the nails when he visited our hospital. We initiated oral baricitinib at 2 mg once daily for treatment. During the 6 months of treatment with baricitinib, the patient's condition did not improve (Figure 1). The patient's weight was 13 kg, and the SALT score was 0. Therefore, we decided to start dupilumab therapy at an induced dose of 400 mg, followed by a monthly dose of 200 mg with a monthly follow-up. The patient first reported hair growth over part of the scalp and the eyebrows and eyelashes after 3 months of dupilumab therapy. The pull test result was negative, and the SALT score was 58 (percentage improvement of SALT = 58%), with little hair regrowth in the eyebrows and eyelashes (Figure 2). After 10 months of treatment, the SALT score was 85 (percentage improvement of SALT = 85%) (Figure 3). The patient's eyebrows and eyelashes were essentially fully restored. The pull test result was negative. The allergic rhinitis episodes were less frequent than before. The results of regular blood tests, coagulation function tests, and liver and kidney function tests of the patient were normal during dupilumab treatment. The patient reported no adverse effects.

## Discussion

Dupilumab is a human monoclonal IgG4 antibody that mediates interleukin-4 (IL-4) and interleukin-13 (IL-13) signaling by specifically binding to the common receptor complex IL-4Ra subunit and regulates Th2 inflammatory responses in allergic diseases (8). Clinical trials and case reports confirmed that dupilumab is effective in patients with AA, particularly in patients with high levels of IgE (7). This is based on epidemiological studies that show a strong association between AA and atopy (5, 9), genetic associations between atopy-related genes and AA (e.g., the Th2 cytokine IL-13), significant upregulation of Th2-related immune products in AA scalp, and elevated serum IgE levels in AA patients (including non-atopic individuals) (3, 4, 10). These studies, including our case, suggest that in addition to Th1, Th2-skewing may play an important role in AA. The exact mechanisms remain unclear.

JAK inhibitors bind to JAK, preventing it from binding to and activating STAT and inhibiting the latter's entry into the nucleus to transduce them. Baricitinib, which is considered a dual JAK1/JAK2 inhibitor, may interrupt Th1 cytokine signaling implicated in the pathogenesis of AA and promote recovery. Not all AA patients have a good response to baricitinib. Th2 predominance-induced dysfunction of regulatory T cells (Tregs) in some patients may contribute to the failure of baricitinib treatment. In a mouse model of AD, excess Th2 signaling resulted in decreased numbers of regulatory T cells (Tregs) and increased IL-13 (11). Dupilumab achieves hair regrowth by blocking Th2 and promoting the recovery of Treg function and quantity in severe AA patients with concomitant atopic diseases (12). There is no study showing any effects of baricitinib in Tregs. This may be one of the explanations for some AA patients, such as the case in this study, who are resistant to baricitinib but respond well to dupilumab.

Interestingly, AA-like reactions have been reported in several patients after the treatment of AD with dupilumab (13–15), which shared many histological features with anti–TNF-a inhibitor-induced psoriatic alopecia. Thus, an imbalance in the cytokine profiles under Th2-blocking might contribute to the development of AA-like reactions, possibly as paradoxical reactions. This suggested a complication in immune networks in the pathogenesis of AA.

The FDA approved dupilumab for the treatment of pediatric patients older than 6 years with moderate to severe AD in 2017 and then expanded the approval to patients older than 6 months in 2022. However, little is known about the potential role of dupilumab in the treatment of pediatric AA, especially at ages younger than 6. In this case, the 4-year-old patient was not satisfied with the efficacy after using baricitinib for 6 months and achieved good efficacy after switching to dupilumab. This shows that high levels of IgE are not prerequisites for dupilumab's successful treatment response. Consequently, clinicians can consider starting pediatric patients with AA on dupilumab even without IgE elevation at baseline. To our knowledge, this is the first report of successful therapy with dupilumab in a patient younger than 6 years who was resistant to JAK inhibitors. This suggests that for pediatric AA patients resistant to JAK inhibitors with or without a history of allergic disease or high IgE, dupilumab may be an effective and safe treatment.

## Data availability statement

The original contributions presented in the study are included in the article/supplementary material, further inquiries can be directed to the corresponding author.

## Ethics statement

The studies involving human participants were reviewed and approved by Ethics Committee of Chongqing Hospital of Traditional Chinese medicine. The patients' legal guardians provided their written informed consent to participate in this study. Written informed consent was obtained from the patient legal guardian for the publication of this case report.

## Author contributions

LC: Writing—original draft. YW: Writing—review and editing. MZ: Writing—review and editing. JZ: Writing—review and editing. XT: Writing—review and editing. ML: Writing—review and editing.
